# Investigation of phylogenetic relationships within *Saxifraga diversifolia* complex (Saxifragaceae) based on restriction‐site associated DNA sequence markers

**DOI:** 10.1002/ece3.10675

**Published:** 2023-11-02

**Authors:** Rui Yuan, Jiaxin Li, Xiaolei Ma, Zhilin Feng, Rui Xing, Shilong Chen, Qingbo Gao

**Affiliations:** ^1^ Key Laboratory of Adaptation and Evolution of Plateau Biota, Northwest Institute of Plateau Biology & Institute of Sanjiangyuan National Park Chinese Academy of Sciences Xining China; ^2^ University of Chinese Academy of Sciences Beijing China; ^3^ Qinghai Provincial Key Laboratory of Crop Molecular Breeding, Northwest Institute of Plateau Biology Chinese Academy of Sciences Xining China

**Keywords:** phylogeny, RAD‐seq, *Saxifraga*, *S. diversifolia* complex, SNP loci

## Abstract

Subsect. *Hirculoideae* Engl. & Irmsch., belonging to *Saxifraga* sect. *Ciliatae* Haw., has high species richness. It can be divided into *S. diversifolia*, *S. pseudohirculus*, and *S. sinomontana* complexes based on morphological characteristics. The species with prominent leaf veins on the posterior leaf edge were placed in the *S. diversifolia* complex, which is mainly distributed on the eastern and southern margins of the Qinghai‐Tibetan Plateau. In this study, 53 samples, representing 15 of the 33 described species in the *S. diversifolia* complex, were sequenced using the Restriction‐site Associated DNA Sequence (RAD‐seq) technique. A total of 111,938 high‐quality SNP loci were screened to investigate the phylogenetic relationships within the *S. diversifolia* complex. The result of the neighbor‐joining (NJ) tree shows that the *S. diversifolia* complex is a paraphyletic group. Despite of some inconsistencies as revealed by genetic structural analysis, clustering results of representative species reconstructed by both NJ and principal component analysis analyses support previous biogeographic and morphological evidences. In addition, long‐distance gene flow events for 11 taxa were detected in the *S. diversifolia* complex, respectively from *S. implicans* 1 to *S. implicans* 2, *S. diversifolia* and *S. maxionggouensis*, and from *S. maxionggouensis* to *S. nigroglandulifera*. These findings may improve our comprehension of the phylogeny, classification, and evolution of the *S. diversifolia* complex.

## Graphical abstract

This paper uses RAD‐seq technology to search for abundant SNP sites. Based on these markers, the phylogenetic relationship of the *Saxifraga diversifolia* Complex (Saxifragaceae) was studied.

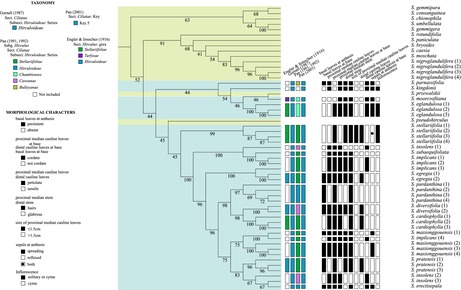

## 1 INTRODUCTION

As common elements in mountainous regions throughout the Northern Hemisphere, *Saxifraga* L. s.str. is dominated by cold‐adapted perennial herbs (Tkach et al., 2015). Also a few circumpolar species, and some extending southwards down the Rocky Mountain/Andean Cordillera (Ebersbach, Muellner‐Riehl, et al., 2017; Gao et al., 2015; Tkach et al., 2015). It has 450 to 500 species all over the world, of which nearly 216 inhabit in China and a total of 139 species are endemic (Pan et al., 2001). These species in China are primarily distributed in the Qinghai‐Tibetan Plateau (QTP), frequently occurring in forest margins, grasslands, and rock faces (Pan et al., 2001). *Saxifraga* has been of great interest in evolutionary studies due to its great variability in morphology and habitat (Abbott & Comes, 2003; Gao et al., 2015; Healy & Gillespie, 2004; Tkach et al., 2015).

There have been many scholars who devoted themselves to clarify the genetic relationship of *Saxifraga*, as well as to revise its taxonomic system. After Linnaeus's original description of *Saxifraga* in 1753, Haworth (1812, 1821) created the sect. *Ciliatae* Haw. depending on the stem, leaf, and corolla. Subsequently, Engler and Gornall attempted to set up infra‐sectional classification of sect. *Ciliatae* with grex and subsection, the former being used as a rank in the older literature (Engler & Irmscher, 1916/1919; Gornall, 1987). Taking account of *Saxifraga* in China, Pan (1991, 1992) adopted a novel approach to the classification of the species by focusing heavily on ovary position, petal morphology, the number of calloses, and veins. In the meantime, he subdivided the sect. *Ciliatae* into four subsections, namely subsect. *Gemmiparae* Engl. & Irmsch., subsect. *Rosulares* Gornall., subsect. *Flagellares* (Clarke) Engl. & Irmsch and subsect. *Hirculoideae* Engl. & Irmsch. This classification scheme was considerably altered in the Flora of China (FOC) by Pan et al. (2001), who replaced the adopted sections with seven informal Keys. Molecular phylogenetic studies have confirmed that sect. *Ciliatae* as delimited by Engler and Irmscher (1916/1919) and Gornall (1987) was monophyletic, in which the subsect. *Hirculoideae* was alonely clustered on a clade with high resolution (Gao et al., 2015; Tkach et al., 2015; Yuan et al., 2023; Zhang et al., 2008). Subsect. *Hirculoideae* could be divided into *S. diversifolia*, *S. pseudohirculus*, and *S. sinomontana* complexes based on their morphological characteristics. That complex was also an informal taxonomic rank corresponding to the series below section or subsection. As an Important multi‐species group, the *S. diversifolia* complex comprises ca. 33 accepted species (Pan et al., 2001). In detail, 29 species and four varieties of *S. diversifolia* complex were recorded and described in the FOC, while four species and one variety were included without descriptive information (Ku, 1989; Pan, 1978; Pan et al., 2001; Zhuang, 2001). Those species with the specific morphological combination were placed in the *S. diversifolia* complex, which corresponds to the Key 5 in the FOC. That is Herbs perennial, mostly tall, solitary; stem usually glandular villous; basal leaves caducous or persistent at anthesis, cauline leaves petiole or sessile, mostly ovate or ovate‐cordate, the submarginal vein running from proximal to distal ends; cyme corymbose; petals usually yellow, spotted or not, two to eight callose; ovary mostly subsuperior (Pan, 1991, 1992; Pan et al., 2001). *S. diversifolia* complex concentrates in the damp woodland and shrubs on the eastern and southern margins of the QTP (Ma et al., 2022). According to the geographical distribution patterns, the *S. diversifolia* complex could be divided into three clades: Himalayan clade, Mountains Around Sichuan Basin clade, and Hengduan Mountains clade, and all of these geographic clades have relatively stable morphological differentiation (Ma et al., 2022). At around 2.12 Ma, Subsect. *Hirculoideae* experienced rapid evolutionary radiations, while the *S. diversifolia* complex underwent a shorter period, leading to low genetic differentiation among these taxa (Gao et al., 2015). However, they did not elaborate on the phylogenetic relationship and morphological evolution trend in the *S. diversifolia* complex.

With the advent of next‐generation sequencing, the restriction‐site associated DNA sequencing (RAD‐seq) has been developed as a simplified genome sequencing technology (Andrews et al., 2016; Davey & Blaxter, 2010; Miller et al., 2007). This method can reduce the complexity of target genomes by relying on enzymatic techniques with or without a reference genome. Since the restriction enzyme cutting sites are widely dispersed throughout the entire genome, massive single nucleotide polymorphism (SNP) can be obtained by RAD‐seq. In comparison with whole genome sequencing, RAD‐seq can analyze multiple samples simultaneously at fairly low costs, and it is suitable for studying high‐density genetic map construction, genome sequence assembly, population evolutionary investigation, as well as phylogenetic research, and so on (Baird et al., 2008; Casanova et al., 2021; Feng et al., 2020; Peterson et al., 2012). This technique has proved particularly useful for phylogenetic reconstruction of infra/intra‐species, especially in genetically similar individuals (e.g., Wang, Ye, et al., 2017; Yang et al., 2016, 2017).

By combining detailed genetic analysis with morphological traits, we hope to shed light on the phylogenetic relationship in the *S. diversifolia* complex. Firstly, take advantage of SNPs obtained from RAD‐seq, we (1) constructed the neighbor‐joining (NJ) tree with unrooted and rooted, and subsequently examined morphological characteristics and earlier taxonomic systems on the phylogeny. (2) Secondly genetic structure and principal component analysis (PCA) were performed based on the SNPs dataset to verify the clustering results of the NJ tree. Finally, (3) potential gene flow was evaluated in order to explore possible genetic communication events. The findings of this study could help us better understand the phylogeny, classification, and evolution of the *S. diversifolia* complex.

## 2 MATERIALS AND METHODS

### 2.1 Taxon sampling

**FIGURE 1:**
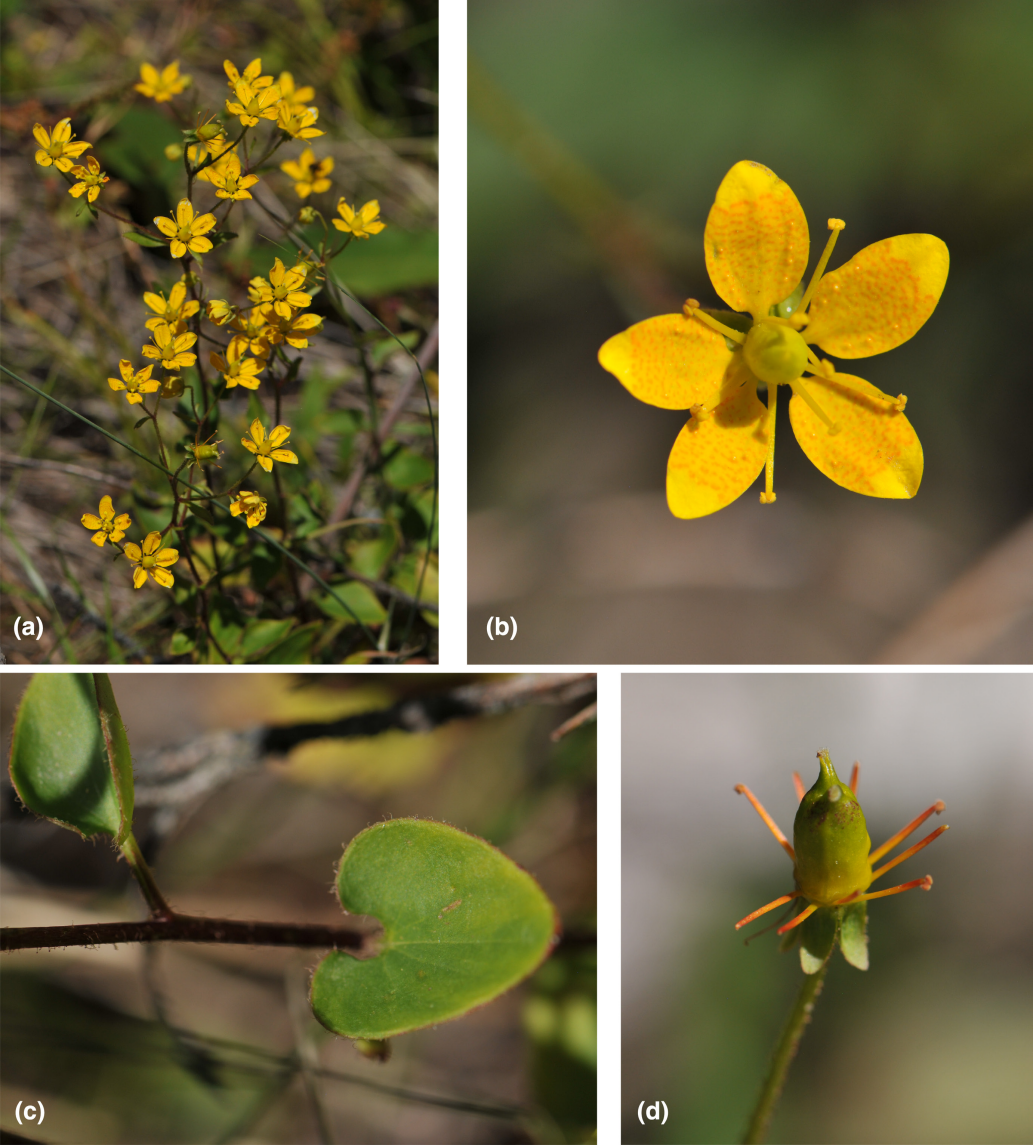
The illustration from *S. egregia*. (a) Herbs perennial, 9–32 cm tall. (b) Sepals yellow with callose, elliptic to ovate, apex obtuse or subacute, base clawed. (c) Cauline leaves cordate‐ovate to cordate, marginally brown villous, base petiolate. (d) Stamens 10; styles 2; ovary subsuperior; sepals reflexed.

A total of 53 samples representing unique 28 species of *Saxifraga*, including 15 of the 33 described *S. diversifolia* complex, were utilized in this investigation. These samples involved subsect. *Hirculoideae* (17 samples), subsect. *Rosulares* (two samples), subsect. *Gemmiparae* (one sample), subsect. *Flagellares* (one sample) in sect. *Ciliatae*, and subsect. *Oppositifoliae* (Hayek) Gornall (one sample), subsect. *Nutantes* Engl. & Irmsch (one sample). subsect. *Aizoonia* (Tausch) Schott (one sample) in sect. *Ligulatae* Haw, and subsect. *Kabschia* (Engl.) Rouy & Camus (one sample) in sect. *Porphyrion* Tausch, subsect. *Triplinervium* (Gaudin) Gornall (one sample) in sect. *Saxifraga*, as well as the sect. *Trachyllum* (Gaudin) W. D. J. Koch (one sample), sect. *Cotylea* Tausch (one sample). For the genetic study, all species other from the *S. diversifolia* complex were employed as outgroups. The majority of our materials were derived from Hengduan mountain in the northwest of Yunnan, China, and the Himalayan in the south of Tibet, China. In addition, five species (*S. bryoides* L., *S. caesia* L., *S. moschata* Wulfen, *S. paniculata* Mill. and *S. rotundifolia* L.) were collected from the European Alps. The young, healthy leaves were collected in the field and dried in silica gel in order to do subsequent sequencing work. The taxon, locality, voucher information, and geographical distribution information are listed in Table 1. As a widespread representative taxon of the *S. diversifolia* complex, pictures of *S. egregia* Engl. are illustrated in Figure 1a–d. Voucher specimens are deposited in the herbarium of Northwest Institute of Plateau Biology (HNWP), Xining, Qinghai, P. R. China, and the University of Leicester, England, UK.

**TABLE 1:** The origin of materials, including the information of taxon, locality, geographic information (latitude, longitude, altitude), voucher and gene flow taxa.

Taxon	Locality	Latitude/N	Longitude/E	Altitude/m	Voucher	Gene flow
***Saxifraga* L**						
**Sect. *Ciliatae* Haw.**						
**Subsect. *Hirculoideae* Engl. & Irmsch.**						
***Saxifraga diversifolia* Complex**						
*S. cardiophylla* Franch. (1)(2)(3)	Muli, Sichuan, China	28.125278	101.1616667	3770	chen03107	*S. cardiophylla*
*S. diversifolia* Wall. ex Ser. (1)(2)	Yanyuan, Sichuan, China	27.685783	101.2232	3240	chen2013522	*S. diversifolia*
*S. eglandulosa* Engl. (1)(2)(3)	Cuona, Xizang, China	27.926361	91.87308333	4460	chen2014461	*S. eglandulosa*
*S. egregia* Engl. (1)	Aba, Sichuan, China	32.767139	101.6669167	3450	chen2014113	
*S. egregia* Engl. (2)	Yajiang, Sichuan, China	30.160806	100.6793889	4290	chen06289	
*S. erectisepala* J. T. Pan	Lijiang, Yunnan, China	27.047333	100.1946944	3570	chen06154	
*S. implicans* H. Smith (1)(2)	Deqin, Yunnan, China	28.339	99.08311111	4310	chen06228	*S. implicans* 1
*S. implicans* H. Smith (3)(4)	Xiangcheng, Sichuan, China	29.129583	99.97541667	3910	chen06263	*S. implicans* 2
*S. insolens* Irmsch. (1)	Xianggelila, Yunnan, China	27.632667	99.67497222	3580	chen2012115	
*S. insolens* Irmsch. (2)(3)	Lijiang, Yunnan, China	27.036667	100.2166667	3070	chen03147	*S. insolens*
*S. kingdonii* C. Marquand	Yadong, Xizang, China	27.622997	89.10879722	4771	chen2013469	
*S. maxionggouensis* J. T. Pan (1)	Xianggelila, Yunnan, China	27.850278	99.07694444	3590	chen03170	
*S. maxionggouensis* J. T. Pan (2)(3)(4)	Xianggelila, Yunnan, China	27.573333	99.80555556	3260	chen03168	*S. maxionggouensis*
*S. moorcroftiana* Wall. ex Sternb.	Yadong, Xizang, China	27.632111	89.03527778	4200	chen2014510	
*S. pardanthina* Hand.‐Mazz. (1)(2)(3)(4)	Xiangchegn, Sichuan, China	29.083972	99.66555556	3380	chen06243	*S. pardanthina*
*S. parnassifolia* D. Don	Nielamu, Xizang, China	28.088611	85.99916667	3320	chen2007096	
*S. pratensis* Engl. & Irmsch. (1)(2)(3)	Xianggelila, Yunnan, China	27.577861	99.86586111	3450	chen06199	*S. pratensis*
*S. stellariifolia* Franch. (1)(2)(3)(4)	Maoxian, Sichuan, China	31.6635	103.9371944	3500	chen06089	*S. stellariifolia*
*S. subaequifoliata* Irmsch.	Longzi, Xizang, China	28.654439	93.40569444	4070	chen2013265	
***S. sinomontana* complex**						
*S. przewalskii* Engl.	Guide, Qinghai, China	36.301489	101.6022083	3662	zhang2016136	
***S. pseudohirculus* complex**						
*S. pseudohirculus* Engl.	Guide, Qinghai, China	36.301489	101.6022083	3662	zhang2016137	
**subsect. *Rosulares* Gornall**						
*S. umbellulata* Hook. f. et Thoms.	Longzi, Xizang, China	28.598039	92.92791111	4010	chen2013298	
*S. gemmigera* Engl.	Seda, Sichuan, China	32.509667	100.3894444	4360	chen2014163	
**Subsect. *Gemmiparae* Engl. & Irmsch.**						
*S. gemmipara* Franch.	Yanyuan, Sichuan, China	27.432969	101.0712083	2141	chen2013529	
**Subsect. *Flagellares* (C. B. Clarke) Engler & Irmscher**						
*S. consanguinea* W. W. Smith	Shiqu, Sichuan, China	33.139944	97.49747222	4570	chen2014247	
**sect. *Ligulatae* Haw.**						
**Subsect. *Aizoonia* (Tausch) Schott**						
*S. paniculata* Mill.	Lago Bianco, Passo Bernina,	46.4078	10.02137	2276	42B	
	Switzerland					
**Subsect. *Oppositifoliae* (Hayek) Gornall**						
*S. chionophila* Franch.	Leiwuqi, Xizang, China	31.084444	96.41222222	4410	chen2007056	
**Subsect. *Nutantes* Engl. & Irmsch.**						
*S. nigroglandulifera* Balakr. (1)(2)(3)	Daocheng, Sichuan, China	29.141528	100.0361111	4610	chen2012196	*S. nigroglandulifera*
*S. nigroglandulifera* Balakr. (4)	Chaya, Xizang, China	30.686389	97.26166667	4090	chen2007195	
**Sect. *Trachyllum* (Gaudin) W. D. J. Koch**						
*S. bryoides* L.	Kalser Torl, path to Granatspitze, Austria	47.11917	12.62307	2529	48A	
**Sect. *Porphyrion* Tausch**						
**Subsect. *Kabschia* (Engl.) Rouy & Camus**						
*S. caesia* L.	East of the Gasthaus, before Albula Pass, Switzerland	46.58792	9.86974	2250	45B	
**Sect. *Saxifraga***						
**Subsect. *Triplinervium* (Gaudin) Gornall**						
*S. moschata* Wulfen	Konigsleiten, Austria	47.26262	12.09061	2253	47C	
**Sect. *Cotylea* Tausch**						
*S. rotundifolia* L.	Val di Loriva, Italy	45.8257	10.61749	1900	34B	

*Note*: The number following the species name denotes the count of individuals.

### 2.2 DNA extraction, RAD‐seq library preparation, and sequencing

Total genomic DNA was extracted from each sample using the DNA secure Plant Kit (Tiangen Biochemical Technology Co., Ltd.) following the manufacturer's standard protocol. Genomic DNA purity and concentration were detected by NanoPhotometer spectrophotometer (IMPLEN) and Qubit 2.0 fluorometer (Life Technologies), respectively. The RAD library preparation was conducted after the protocol described by Baird et al. (2008). In brief, genomic DNA from each qualified sample was digested by *Eco*RI (New England Biolabs), and an adapter (P1) was ligated to the fragment's compatible ends. The adapter‐ligated fragments were subsequently pooled, randomly sheared, and size‐selected. Then DNA was ligated to a second adapter (P2), a Y adapter that has divergent ends, which allowed selective amplification of RAD tags containing both P1 and P2 adapters to complete the library construction. The library with high quality and yield was sequenced by Illumina HiSeq PE150. The raw sequencing data contains splice information, low‐quality bases, and undetected bases (expressed as N), which can significantly obstruct the following analysis. Therefore, it is necessary to perform quality control on the raw data with the subsequent filtering conditions by fastp v.0.23.1 (Chen et al., 2018): (1) the N content in any read was greater than 10% of the base; (2) the number of low quality (*Q* < = 5) bases in any read exceeded 50%; and (3) any read contained the adapter content; if so, the paired reads were removed (Yan et al., 2013).

### 2.3 SNP calling

In this study, a de novo assembly of reference genome was performed from *S. sinomontana* J‐T. Pan & Gornall genome survey data using SOAPdenovo2 (Luo et al., 2012). The filtered clean data was mapped to the reference genome by BWA v.0.7.17 with the parameter mem (Li & Durbin, 2009). Next, SNP calling for 53 samples was done using SAMtools and BCFtools v.1.9 (Danecek & McCarthy, 2017; Li et al., 2009). The initial filtering of polymorphic loci in the sample relied on Q20 quality control and the distance between SNP loci. That is, the SNP loci were eliminated if the error rate exceeded 1%, and both SNPs were removed if the gap was less than five base pairs, which was regarded as sequencing, experimental, or analytical error due to its extremely low likelihood. However, the deeper filtering was required to guarantee high quality for avoid calling false‐positive SNPs. VCFtools v.0.1.16 (Danecek et al., 2011) was used to again screen SNPs concerning two filtering options: maximum missing genotype greater than 0.4, and minimum mean read depth less than 2. Loci that failed to meet the quantification threshold for any of the filtering criteria were removed and excluded from the following analyses.

### 2.4 Phylogenetic analysis, taxonomic, and morphological researches

To demonstrate the affinities between the *S. diversifolia* complex engaged in this study, we constructed a phylogenetic tree based on the filtered SNPs dataset. Briefly, TreeBeST v.1.9.2 was applied to calculate the genetic distance matrix between individuals, and then the unrooted and rooted trees were established by the NJ method (Vilella et al., 2009). The bootstrap value was determined after 1000 iterations, which was used to evaluate the reliability of the tree topology. Eventually, the NJ trees were visualized and embellished using the online tool ITOL v.6 (Letunic & Bork, 2007). The rooted phylogenetic tree was compared with the taxonomic treatments of Engler and Irmscher (1916/1919), Gornall (1987), Pan (1991, 1992), and Pan et al. (2001). For the research of morphological characteristics, we focused on critical characters that had been used in previous studies to delineate the *S. diversifolia* complex. On the one hand, some characteristics were crucial for the traditional classification, as well as being used more frequently. On the other hand, several were previously undervalued in the *S. diversifolia* complex. In detail, the studied characters were the basal leaves at anthesis (persistent or absent), proximal median or distal cauline leaves (petiolate or sessile), basal leaves at base (cordate or not cordate), proximal median or distal cauline leaves at base (cordate or not cordate), proximal median or distal stem (hairs or glabrous), size of proximal median cauline leaves (≤1.5 cm or >1.5 cm), sepals at anthesis (spreading, reflexed, or both), inflorescence (solitary or cyme, cyme). Morphological data used in this study were taken from the monographs mentioned above and literature descriptions (Engler & Irmscher, 1916/1919; Ma et al., 2022; Pan, 1991, 1992; Pan et al., 2001).

### 2.5 Principal component and genetic structure analysis

The PCA has been a useful tool for genetic data analysis since it was first introduced by Menozzi et al. (1978). PCA analysis can display the results of sample clustering, and infer the evolutionary relationships between populations (Novembre & Stephens, 2008; Patterson et al., 2006; Reich et al., 2008). In this study, we first used PLINK v.1.9 to convert the VCF file storing SNPs into the input file required by GCTA v.1.94.1 (Purcell et al., 2007; Yang et al., 2011). Furthermore, GCTA was utilized to calculate the eigenvalues and eigenvectors. We visualized the PCA clustering results with R v.4.0.2 package (ggplot2), which contained two‐dimensional and three‐dimensional graphs (https://www.r‐project.org; Ginestet, 2011). At the same time, the genetic structure of 53 samples was constructed using ADMIXTURE v.1.3.0 (Alexander et al., 2009). Assuming that the samples under study originated from *K* different ancestors, the proportion of each hypothetical ancestor in the genetic composition of each sample was analyzed. In order to calculate the error rate, we employed fivefold cross‐validation with *K* values ranging from one to eight for 10 iterations. The optimal number of clusters was determined by using the *K* value that corresponded to the minimum cross‐validation error, and then the genetic structure was drawn by R.

### 2.6 Gene flow evaluation

The movement of genes from one population into another gene pool is referred to as gene flow or gene migration, and it changes the gene frequency of the recipient (Ashley, 2010; Semizer‐Cuming et al., 2017). It is the crucial source of genetic variation, influencing the genetic diversity of populations and even generating novel trait fusions. In this paper, TreeMix analysis was performed to test whether there is potential gene flow in the speciation of *S. diversifolia* complex. A total of 11 taxa with identical collection locality and at least two individuals were retained to investigate the gene flow phenomenon (Table 1). It is worth mentioning that two individuals of *S. implicans* H. Smith were respectively collected from Yunnan and Sichuan, China, and thus designated as two separate populations. We used VCFtools to filter out missing data from the SNPs dataset (parameter: max‐missing 1) and to remove linkage disequilibrium loci by PLINK v.1.9 (parameter: indep‐pairwise 50 10 0.2) (Purcell et al., 2007). The above‐filtered dataset was treated as the input file for TreeMix v.1.13, which was applied to infer the specified number of gene migration events (Fitak, 2021). The software calculated the actual covariance between each pair of populations based on allele frequencies, as well as building a maximum likelihood tree to determine phylogenetic relationships to count the estimated covariance. The magnitude of the difference between the actual and estimated values was used to judge whether gene flow occurred between populations. We set *m* to 1–10, assuming there will be 1–10 gene migration events in the group under study, and then used the R tool (OptM) to search for the optimal *m* value. In the end, the ideal results were visualized using Treemix's built‐in R script.

## 3 RESULTS

### 3.1 Sequencing results and variant detection

The sequencing of the 53 samples that passed quality control is shown in Appendix Table S1. Raw data range from 1,055,962,800 to 8,952,689,700 bp, and filtered clean data vary from 1,041,601,800 to 7,827,153,300 bp. The average error rate of sequencing results is 0.03, meanwhile, Q20 and Q30 of all samples are 96.45% and 91.2% respectively. A range of 37.21% to 39.48% GC is also present. Then, each sample was mapped separately to the reference genome, and Appendix Table S2 provides important information of mapping rate, average depth, and coverage. After eliminating duplicates, the mapping result displays a total of 977,338,682 bp from 53 samples, with an average mapping rate of 77.89%, average sequencing depth of 9.55, and average coverage of 10.92% for at least one base. Both homogeneity and similarity between the sample and the reference genome are indicated by these metrics. The mapping result satisfied the criteria for re‐sequencing analysis, which also provides reliable data to support the next phylogenetic analysis. Subsequently, SAMTOOLS identified a total of 900,665 SNP loci, and 111,938 high‐quality SNP loci are detected after a series of filtering procedures.

### 3.2 Phylogenetic analysis, taxonomic and morphological studies

**FIGURE 2:**
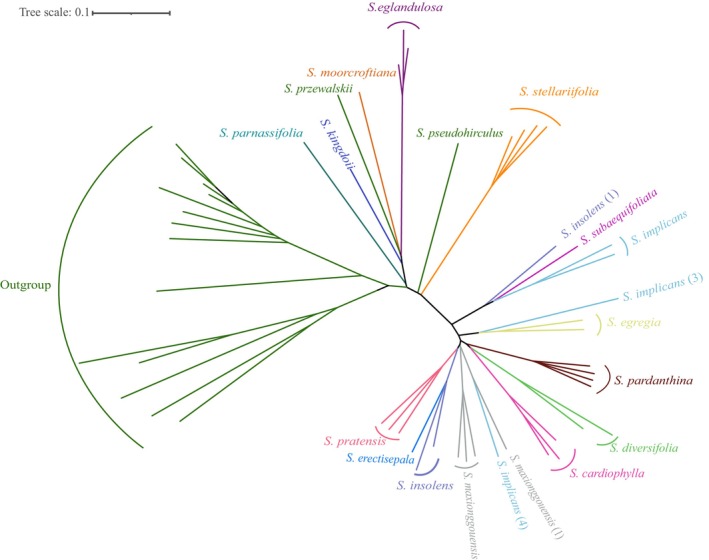
Phylogenetic tree reconstructed based on the SNP data of high quality using unrooted neighbor‐joining (NJ) methods. Taxa includes 53 samples representing 28 species of *Saxifraga*, including 15 of the 33 described in the *S. diversifolia* complex.

**FIGURE 3:**
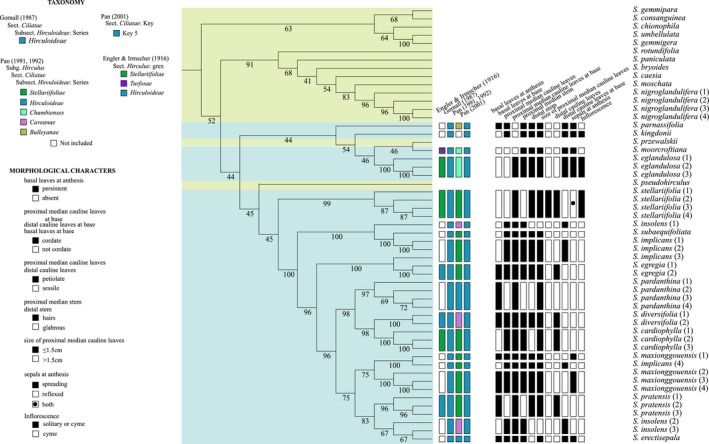
Phylogenetic tree reconstructed based on the SNP data of high quality using rooted neighbor‐joining (NJ) methods. Taxa includes 53 samples representing unique 28 species of *Saxifraga*, including 15 of the 33 described in the *S. diversifolia* complex. Numbers at the nodes represent bootstrap values of the support rate. Different colors represent different groups, meaning that green indicates outgroups and blue denotes *S. diversifolia* complex. The taxonomic information in previous studies and important morphological characteristics of the *S. diversifolia* complex are arranged in columns.

High‐quality SNP data were utilized to construct NJ phylogenetic trees, including unrooted and rooted (Figures 2 and 3). The phylogenetic results show that majority of the outgroup species clustered outside the *S. diversifolia* complex as a separate clade with lower support, except for *S. pseudohirculus* Engl. and *S. przewalskii* Engl. (Figures 2 and 3). Voucher scans of *S. pseudohirculus* and *S. przewalskii* are displayed in Appendix Figure S1. Of these two species, the former belonged to the *S. pseudohirculus* complex, and the latter was classified into the *S. sinomontana* complex. The three complexes mentioned are above all affiliated to subsect. *Hirculoideae* as delimited by Pan et al. (2001). Our results demonstrate that most members of the same taxon are typically found clustered together (Figure 2). However, there are certain exceptions, such as, *S. implicans*, *S. insolens* Irmsch. and *S. maxionggouensis* J. T. Pan, three taxa in which at least one individual has been clustered out. The clustering results of the rooted NJ tree are shown in Figure 3, whereas some of its nodes lack support, especially in the backbone. Specifically, five taxa were poorly supported by clustering on a branch, namely *S. kingdonii* C. Marquand, *S. parnassifolia* D. Don, *S. moorcroftiana* Wall. ex Sternb. and *S. eglandulosa* Engl. in the *S. diversifolia* complex and *S. przewalskii* in the outgroup. Another species in the outgroup, *S. pseudohirculus*, also intermixes with members of the *S. diversifolia* complex, although this result is as well not backed up by the high bootstrap value. In this case, *S. pseudohirculus* does not form sister relationship with the other species, but rather branches out on its own. Four individuals of *S. stellariifolia* Franch. with high support (bootstrap: 99) clustered together on a branch (Figure 3). In addition, the remaining species of the *S. diversifolia* complex contained 27 samples representing 10 taxa, all of which are high support (bootstrap: 100) grouped in a branch (Figure 3). These taxa are composed of *S. insolens*, *S. subaequifoliata*, *S. implicans*, *S. egregia*, *S. pardanthina*, *S. diversifolia*, *S. cardiophylla*, *S. maxionggouensis*, *S. pratensis*, and *S. erectisepala*. However, there is insufficient support for the sisterhood formed by this group of 27 samples and the branch comprising *S. stellariifolia*. On the classification of the *S. diversifolia* complex, Engler and Irmscher (1916/1919), Gornall (1987), Pan (1991, 1992), and Pan et al. (2001) provided different solutions for dealing with it (Figure 3). Gornall (1987) and Pan et al. (2001) accommodated these species within Series *Hirculoideae* in Subsect. *Hirculoideae* and Key 5 in Sect. *Ciliatae*, respectively, for the *S. diversifolia* complex species involved in this study. The majority of the investigated taxa were not included in Engler and Irmscher (1916/1919)'s taxonomic system. Among the species involved, except for *S. moorcroftiana*, which was classified into grex. *Turfosae* Engl. et Irmscher, the rest either belong to the grex. *Stellariifoliae* Engl. et Irmscher or the grex. *Hirculoideae* Engl. et Irmscher, and all three ranks being in the Sect. *Hirculus*. In contrast, Pan (1991, 1992)'s taxonomic system defined the maximum number of divisional ranks. He categorized these taxa into five series of Subsect. *Hirculoideae*, that is, Ser. *Stellariifoliae* (Engl. et Irmsch.) J. T. Pan, Ser. *Hirculoideae* (Engl. et Irmsch.) Gornall, Ser. *Chumbienses* J. T. Pan, Ser. *Caveanae* J. T. Pan, Ser. *Bulleyanae* J. T. Pan.

To varying degrees, some of the morphological characters traditionally used to identify *S. diversifolia* complex are insufficient to distinguish the lineages determined in the molecular phylogenetic study. On the contrary, several underestimated characteristics seem to play a role in determining the taxonomic units of this group.

#### 3.2.1 Basal leaves at anthesis

Whether the presence of basal leaves at anthesis is an important identifying feature of the *S. diversifolia* complex in FOC. *S. kingdonii*, *S. moorcroftiana*, *S. eglandulosa*, and *S. stellariifolia* essentially lose their basal leaves during the time of flowering, except for *S. parnassifolia*. This feature is not readily apparent in the lineage comprising the remaining 27 samples, meaning that some species have basal leaves, while other have the opposite (Figure 3).

#### 3.2.2 Basal, proximal median, and distal cauline leaves at base

The shape of the leaf base is varied, such as cordate, rounded, and obtuse. Therefore, leaf base is frequently utilized as a key characteristic to differentiate taxa in traditional taxonomy. The basal and proximal median cauline leaves of the *S. diversifolia* complex involved in this study have more cordate bases. In the *S. stellariifolia* and most taxa of the lineage representing 27 samples, the distal cauline leaves at the base are not cordate, whereas the situation is the opposite in *S. parnassifolia*, *S. kingdonii*, *S. moorcroftiana*, and *S. eglandulosa* (Figure 3). Thus, the base shape of distal cauline leaves may contribute to the differentiation of the lineages of the *S. diversifolia* complex.

#### 3.2.3 Petiole of proximal median and distal cauline leaves

The petiole has occasionally been employed as a distinguishing characteristics to pinpoint species and circumscribe sections or series. The different parts of cauline leaves involved in this study are petiole or sessile, which have different conditions (Figure 3). The majority of species are petiole for proximal median cauline leaves, in addition to the *S. parnassifolia*, *S. kingdonii*, and *S. moorcroftiana*. In the *S. diversifolia* complex, it has always been observed that the petiole of cauline leaves gradually smaller from the bottom to the top, as well as sessile. In this paper, *S. parnassifolia*, *S. kingdonii*, *S. moorcroftiana*, and *S. eglandulosa* all share the common feature of having sessile distal cauline leaves. But *S. stellariifolia* has petiole of distal cauline leaves about 0.2–1 cm long. In the lineage representing 27 samples, the distal cauline leaves of species with petioles or sessile are intermixed.

#### 3.2.4 Hairy of proximal median and distal stem

At least the proximal stem nodes and petiole bases are covered with brown, crisped, villous hairs, which is a significant morphological character in Subsect. *Hirculoideae* (Engler & Irmscher, 1916/1919). Most members of *S. diversifolia* complex show more or less hair stem, either at the proximal median or distal stem (Figure 3). However, the stem of *S. insolens* is glabrous by the description in the Flora Reipublicae Popularis Sinicae (FRPS) and FOC.

#### 3.2.5 Size of proximal median cauline leaves

The proximal median cauline leaves of *S. stellariifolia* are less than or equal to 1.5 cm (Figure 3). Compared to other taxa of the *S. diversifolia* complex involved in this study, the proximal median cauline leaves of *S. stellariifolia* look smaller in size. It may be an important morphological trait to help distinguish *S. stellariifolia*.

#### 3.2.6 Sepals at anthesis

The *S. diversifolia* complex has either reflexed or ascended to spreading sepals (Figure 3). At anthesis, the sepals of *S. parnassifolia*, *S. kingdonii*, *S. moorcroftiana*, and *S. eglandulosa* expand outward. This characteristic exists in several species of the lineage composed of 27 samples, yet there are also some species with reflex sepals. However, *S. stellariifolia* has sepals in both of these states.

#### 3.2.7 Inflorescence

Although the inflorescence is closely related to plant taxonomy, it is less effective in the identification of the *S. diversifolia* complex. Most of the species in this group are cyme, with different presentation forms, such as corymbose, conical, and racemose (Figure 3). *S. kingdonii*, *S. eglandulosa*, and *S. stellariifolia* are also noted in the form of flower solitary at the top of the stem.

### 3.3 Principal component and genetic structure analysis

**FIGURE 4:**
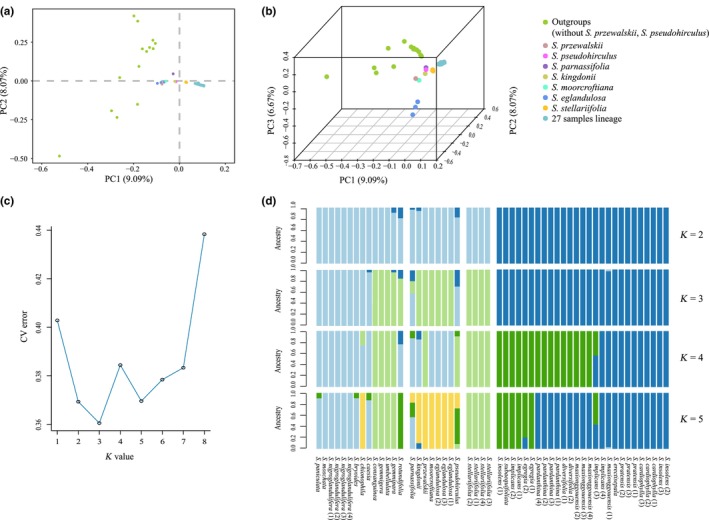
Results of principal component (PCA) and genetic structure analysis. Display the clustering results of PCA in two‐dimensional (a) and three‐dimensional (b). Different colors represent different taxa, where 27 samples lineage stand for 10 taxa, namely *S. insolens*, *S. subaequifoliata*, *S. implicans*, *S. egregia*, *S. pardanthina*, *S. diversifolia*, *S. cardiophylla*, *S. maxionggouensis*, *S. pratensis*, *S. erectisepala*. (c) Different *k* values correspond to the CV error value. The result shows that the CV error value is the minimum when *K* equals three, which is the optimal number of clusters. (d) The clustering results of genetic structure in 53 samples when *k* is equal to two to five.

If two samples are further apart in the PCA, the greater the difference in genetic background between the two samples, and vice versa. In this study, the clustering results of the PCA are presented in different dimensions (Figure 4a,b). The first, second, and third axes of the PCA explained 9.09%, 8.07%, and 6.67% of the variation, respectively. The results show that the outgroup species are scattered in the periphery except for *S. pseudohirculus* and *S. przewalskii*, which is consistent with the NJ tree analysis. Here, *S. przewalskii* is clustered with *S. eglandulosa* and *S. moorcroftiana*, meanwhile *S. pseudohirculus* and *S. kingdonii* are close together (Figure 4a). Four individuals of *S. stellariifolia* remained adjacent to each other without mixing with other species (Figure 4a,b). In addition, the lineage containing 27 samples is obviously classifiable as a group that has explicit genetic differentiation from other taxa of the *S. diversifolia* complex. The results reveal that *S. parnassifolia*, *S. kingdonii*, *S. moorcroftiana*, and *S. eglandulosa* are more near to the outgroup, indicating that these taxa might have a closer genetic relationship with the outgroup. At the same time, 53 samples were analyzed for genetic structure based on 111,938 SNP loci. By comparing the error rate of cross‐validation with different *K* values, the result shows that the error value of CV is lowest when *K* equals three (Figure 4c). According to the structural analysis, it is best to divide the 53 individuals into three genetic groupings. The genetic structure of the samples with *K* equal to two to five is displayed in Figure 4d. When *K* equals two, *S. parnassifolia*, *S. kingdonii*, *S. moorcroftiana*, *S. eglandulosa*, and *S. stellariifolia* plus the outgroup are combined into one subgroup, while 27 samples lineage forms another. In the case where *K* is set to three, one part of the outgroup is a subgroup, and the other part constitutes a subgroup with *S. kingdonii*, *S. moorcroftiana*, *S. eglandulosa*, and *S. stellariifolia*. The lineage consisting of the remaining 27 samples is still as a separate subgroup. Once *K* increases to four or five, the 27 sample lineage with stable properties begins to be split into two subgroups. Regardless of the value set for *K*, there are mixed samples presented and located predominantly in the outgroup, *S. parnassifolia* and *S. kingdonii*, suggesting that these species may engage in more gene exchange.

### 3.4 Gene flow evaluation

**FIGURE 5:**
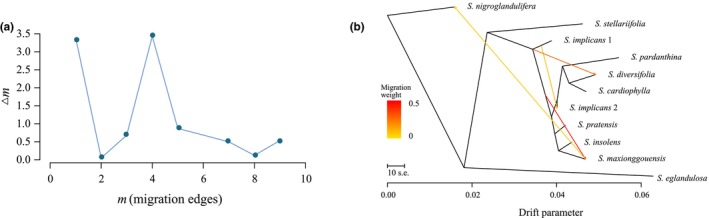
The result of gene flow evaluation. (a) The different migration edges correspond to the change rate of likelihood value (Δ*m*). When the number of gene exchanges is four, it is the relatively optimal migration edge. (b) The direction and weight of four gene flow events. The arrows indicate the direction of gene flow and the legend represents the weights. Each branch represents a taxon and its length refers to the proportion of genetic drift.

A total of 5235 SNPs were retained after removing missing data and linkage disequilibrium loci in order to assess gene flow events. The potential for gene communication among 11 taxa was investigated in this study. The relative optimal *m* value within the specified range was judged by using the change rate of likelihood value. To put it another way, *m* is at its best when Δ*m* achieves its greatest value. When the number of displayed gene exchanges is four, it is the best migration edge in our study (Figure 5a). In Figure 5b, four gene exchange events are shown along with their directions and weights. Specifically, there was gene flow from *S. implicans* 1 to *S. implicans* 2, *S. diversifolia*, and *S. maxionggouensis*. The latter two communication events account for the greater weight among them. Exchange of genes from *S. maxionggouensis* to *S. nigroglandulifera* has also been detected.

## 4 DISCUSSION

### 4.1 Phylogenetic inferences in the *S. diversifolia* complex

As a relatively young group, Subsect. *Hirculoideae* have undergone rapid evolutionary radiation, and the *S. diversifolia* complex contained within it is no exception (Ebersbach et al., 2018; Ebersbach, Schnitzler, et al., 2017; Gao et al., 2015). It is challenging for scientific classification and accurate identification in the *S. diversifolia* complex due to low genetic differentiation and minor morphological variation. As a result of these factors, the process in terms of resource exploitation and utilization of the complex has slowed down considerably. This study aims to resolve the *S. diversifolia* complex's phylogenetic relationships using RAD‐seq molecular technique. To detect high‐quality and abundant SNPs, we employed rad sequencing data from 53 samples representing unique 28 species of *Saxifraga*, including 15 of the 33 described *S. diversifolia* complex. The resolution of genetic markers would be enhanced by high density SNPs, which may also benefit phylogenetic research in the complex taxa.

The NJ results show that the *S. diversifolia* complex may be a paraphyletic group, because *S. pseudohirculus* and *S. przewalskii* intermingle with this group of species. *S. pseudohirculus* and *S. przewalskii* were respectively classified into the *S. pseudohirculus* complex and the *S. sinomontana* complex, based on the distinction between glandular hairs and pilose on the stem (Pan et al., 2001). However, since the constructed NJ tree lacked the high support of backbone nodes, the clustering position of *S. przewalksii* and *S. pseudohirculus* might change once in some cases of adding species or changing the reference genome. In many of the earlier studies, whether Zhang et al. (2008), Gao et al. (2015) or Tkach et al. (2015), their results suffered from the same absence of strong support. Yuan et al. (2023) reconstructed the phylogenetic relationship of sect. *Ciliatae* with complete chloroplasts. Despite the fact that she highlights subsect. *Hirculoideae*'s monophyly, those three complexes that make up the subsection were not separately clustered on a branch. Therefore, it is demonstrated that none of *S. diversifolia*, *S. pseudohirculus*, and *S. sinomontana* complexes are monophyletic in view of the conclusion of this work and Yuan et al. (2023). Meanwhile, *S. pseudohirculus* and many members of the *S. diversifolia* complex were also highly accepted as being clustered in a clade in her results. However, both morphological and plastid genomic evidences suggested that *S. przewalksii* would be closer to the *S. sinomontana* complex rather than the *S. diversifolia* complex.

Although PCA and NJ conclusions are highly consistent, genetic structure analysis shows some different clustering results. The error value of CV is lowest when *K* equals three, which implies that the division of 53 individuals into three genetic groupings yields the highest confidence level, where the lineage comprising the remaining 27 samples is formed. Similarly, the 27 samples lineage and other samples have clear genetic differentiation in the PCA analysis. Even though some branches of the NJ tree have low bootstrap values, the clustering results of the *S. diversifolia* complex are justified on the basis of biogeographical and morphological evidences. A total of 443 geographical distribution records were used by Ma et al. (2022) to construct the distribution pattern of this complex group. He identified three centers of diversity in the *S. diversifolia* complex and delineated three lineages, namely the Himalayan, the Mountains Around Sichuan Basin, and the Hengduan Mountains Branch. There is a match between the branches mentioned above and the clustering situation in this paper. In detail, *S. kingdonii*, *S. parnassifolia*, *S. moorcroftiana*, and *S. eglandulosa* are all within the Himalayan branch. This branch is primarily found on the southern slopes of the Himalayas, from Nyalam to Cuona counties, with some species also occurring in Nepal (Shinobu & Gornall, 2012). With regard to *S. moorcroftiana* and *S. eglandulosa*, Engler and Irmscher (1916/1919) placed these two species in grex *Turfosae* and *Stellariifoliae*, respectively. However, Pan (1991, 1992) attributed them to the series *Chumbienses* by sepals glandular pilose or pilose, five to seven veins, confluent into a verruca at apex; petals yellow, sometimes with purple spot. This treatment is comparable to the findings of the current study in that *S. moorcroftiana* and *S. eglandulosa* share a lot of genetic similarities. The *S. stellariifolia* is located in the Mountains Around Sichuan Basin branch. In addition, this branch identified by Ma et al. (2022) also includes *S. giraldiana* Engl. These two taxa cover the southern slope of the Qinling Mountains, Shennongjia Forest Region, Emei Mountain, and parts of the western Sichuan Plateau in China. It has a small amount of overlap with the branch species of Hengduan Mountain in southwest Sichuan, China. Engler and Irmscher (1916/1919) and Pan (1991, 1992) shared the same approach to the classification of *S. stellariifolia*, assigning it to the Ser. *Stellariifoliae*. The remaining 27 species are grouped into the clade in this study, corresponding to the Hengduan Mountains branch. Those species are clustered in a separate group at the end of the evolutionary tree. In addition to being in China's northwest Yunnan and southwest Sichuan, they also cross over at southeast Tibet with the branches of the Himalayas' southern slope. Among the 27 samples representing 10 taxa, Engler and Irmscher (1916/1919)'s taxonomy scheme only included *S. egregia*, and *S. pratensis* Engl. & Irmsch., *S. diversifolia* Wall. ex Ser., and *S. cardiophylla* Franch. He proposed that the first three species should be classified as grex. *Hirculoideae* and *S. cardiophylla* as grex. *Stellariifoliae*. These species are classified by Pan (1991, 1992) in further detail as belonging to three series: *Stellariifoliae*, *Caveanae*, and *Hirculoideae*. In contrast, Gornall (1987) and Pan et al. (2001) did not explicitly split the *S. diversifolia* complex and instead included all species in the Ser. *Hirculoideae* or key 5.

### 4.2 Hypothesis of origin and spread in the *S. diversifolia* complex

Molecular evidence indicates that Subsect. *Hirculoideae* has recently differentiated and diverged from other subsections of Sect. *Ciliatae*, which were previously considered to be at the end of the evolutionary tree (Gao et al., 2015; Yuan et al., 2023; Zhang et al., 2008). In the work of Yuan et al. (2023), *S. parnassifolia*, *S. moorcroftiana*, and *S. eglandulosa* were concentrated (Bayesian inference: 1/maximum likelihood: 100) in the first diverged clade in Subsect. *Hirculoideae*. It is therefore speculated that *S. kingdonii*, *S. parnassifolia*, *S. moorcroftiana*, and *S. eglandulosa* could be previously differentiated taxa within the *S. diversifolia* complex. In contrast, the 27 samples lineage might be the most recently differentiated taxon. Subsect. *Hirculoideae* is known to have undergone rapid radiation divergence in the QTP‐Hengduan Mountain around 2.12 Ma, resulting in the present species diversity (Ebersbach, Muellner‐Riehl, et al., 2017; Gao et al., 2015). Species of the *S. diversifolia* complex are probably not alien in origin, but undergo in situ radiation in the QTP region (Ebersbach, Muellner‐Riehl, et al., 2017). The QTP, the highest average altitude region in the world, its south (Himalayas) and the southeast (Hengduan Mountains) constitute major biodiversity hotspots with primary proportion of alpine elements (Mulch & Chamberlain, 2006; Myers et al., 2000; Wen et al., 2014). Several spectacular in situ radiations have been reported, perhaps facilitated by the island‐like distribution of montane and alpine habitats resulting from the uplift of the QTP (Hughes & Atchison, 2015; López‐Pujol et al., 2011; Wen et al., 2014). As one of the most rugged mountain ranges in the world, the role of the Hengduan Mountains cannot be ignored. The physiographic complexity of these mountains appears to be a critical element contributing to rapid radiations through allopatric speciation along with the evolution of narrow endemism (Hughes et al., 2015; Hughes & Atchison, 2015). In addition, Ma et al. (2022) considered that the most widely distributed *S. egregia* occurs in the Hengduan Mountains, Qinghai and Gansu Province in China, resulting from the dispersal process of *S. diversifolia* complex. Our phylogenetic results also show that *S. egregia* is likely to be a later divergent taxon. Thus, we hypothesize that during the process of Sect. *Ciliatae* diverged in the QTP region, the *S. diversifolia* complex of species first differentiating in the southern Himalayan slope region and thereafter dispersing to the Hengduan Mountains and other areas.

### 4.3 Morphological studies in the *S. diversifolia* complex

It is more difficult to find significant traits for classification purposes at a lower taxonomic rank, such as a subsection or series. The complexity of the complicated group in character study is another challenge. In this study, certain significant physical traits of the *S. diversifolia* complex of species were gathered, including the leaf base, hairs on the stems, inflorescences, etc. Combined with the morphological quantitative findings by Ma et al. (2022), the morphological evolution of the *S. diversifolia* complex has followed some patterns and trends. In particular, the plant tends to be dwarfed, the distal leaves become smaller, and there is a clear distinction between the morphology of the proximal and distal leaves. It is possible that the majority of *S. kingdonii*, *S. parnassifolia*, *S. moorcroftiana*, and *S. eglandulosa* live in the forest understorey, where the habitat is rather homogeneous and the wind pressure is relatively low, leading to the development of large leaves with uniform shape up and down. While the members of 27 sample lineage are generally found in shrubs, with the leaves near the top of the scrub gradually evolving sparse, small leaf properties due to the influence of the scrub and outside wind. Among them, the proximal median leaves are less affected, which is similar to the shape of *S. kingdonii*, *S. parnassifolia*, *S. moorcroftiana*, and *S. eglandulosa*. *S. stellariifolia* and *S. giraldiana* included in the Ma et al. (2022), both species with marginal prominent leaf veins that do not run from base to apex, which are obviously different from the other lineages (Zhang et al., 2015). Simultaneously, most of them are often rooted in alpine meadows, resulting in lower stature, numerous and compact leaves, as well as smaller (Pan, 1991, 1992). Gornall (1987) also suggested that leaf variation in *Saxifraga* is somehow related to the species' habitat. In exposed situations, *Saxifraga* often has small leaves that are entire or nearly so, whereas large, deeply lobed leaves are primarily found in sheltered habitats (Webb & Gornall, 1989).

The interpretation of the molecular results in this paper is hampered by some traditional and significant morphological features. The hairs on the stem, as an illustration. There are three different forms of hair on *Saxifraga*: pilose, glandular, and glandular pilose (Webb & Gornall, 1989). The specimen must be carefully observed because the glands are prone to falling off, making accurate information gathering challenging. There is a possibility that glandular hairs and glandular pilose would be regarded as pilose. We therefore focused only on whether the stems were hairy in the *S. diversifolia* complex. Our results show that almost all the species are hairy on different parts of the stem, except for *S. insolens* which is glabrous, but its petiole is proximally brown pilose at the margin. From the functional point of view, these hairs may help shield plants from insect predation or oviposition (Webb & Gornall, 1989).

### 4.4 Gene flow evaluation

Gene flow significantly affects genetic differentiation between populations, and even increases genetic diversity as well as maintains the integrity of species (Abbott et al., 2013). The mathematical theory of population genetics, initially put forth by Haldane and Wright et al. (Slatkin, 1987), revealed gene flow as a potentially important evolutionary force. At present, it is believed that plant gene flow is diverse and idiosyncratic and frequently happens at levels that are evolutionarily significant at distances of hundreds or thousands of meters (Ellstrand, 2014). There is a strong relationship between gene flow and the geographical distance between populations. In other words, the likelihood of gene communication increases with decreasing spatial distance, while there may be little or no gene flow between populations that are far apart (Wang, Ye, et al., 2017). Short‐distance gene communication is more likely to be accompanied by wind‐drifting of plant pollen, insects, or seed dispersal (Garant et al., 2007). It is reported that the flowers of *Saxifraga* of Europe are mainly pollinated by insects, but they are not specialized or apparently adapted to any particular insects (Webb & Gornall, 1989). Among the most frequently recorded visitors are flies and beetles, with bees appearing less frequently. The pollination pathways of *Saxifraga* from the QTP, however, have not yet been thoroughly studied by scholars. Also, no specific mechanism was discovered for *Saxifraga* to spread seeds from the dehiscent capsule. As a result, the seeds of this taxon probably rely on gravity or wind as the medium to spread, which would limit their ability to travel very far. However, we have detected long‐distance gene flow events in the *S. diversifolia* complex, from *S. implicans* 1 to *S. implicans* 2, *S. diversifolia*, and *S. maxionggouensis*, and from *S. maxionggouensis* to *S. nigroglandulifera*. Long‐distance gene flow like that described above appears to be maintained at historical level, as a result of cumulative historical gene flows. Ancestral populations were geographically isolated from each other to form small, mutually fragmented populations due to geological or climatic factors. Limited gene flow or no communication may have occurred between these segregated populations, which gradually led to genetic divergence (Hey, 2006). In this study, the gene flow signatures we discovered might be relevant to the Pleistocene glaciations. The QTP is known that uniform massive ice sheet not to form during the Last Glacial Maximum, leading vegetation over the region to present variable responses (Hewitt, 2004). Jia et al. (2018) revealed that the distribution pattern of *S. egregia* could be explained by the “platform refugia/local expansion” hypothesis, which argues that one or several isolated refugia exist on the QTP, certain cold‐tolerant plants survive on these refugia during glaciations, followed by interglacial or post‐glacial range expansion from nearby refugia (Gao et al., 2016). *S. egregia* underwent range shifts during the ice age, culminating in allopatric divergence among isolated “alpine islands.” Compared to within species, gene flow between different species can greatly enrich the degree of biodiversity in nature and further contribute to species formation (Abbott et al., 2013; Bell & Travis, 2005). Mallet (2007) revealed that approximately 40%–70% of angiosperms contain interspecific gene flow events during their origin and evolutionary history. For example, Arabidopsis thaliana underwent two or three interspecific genome duplication events, one of which was associated with early differentiation in angiosperms (Bowers et al., 2003). Although only a few gene flow phenomena, such as hybridization, have been documented in the sect. *Ciliatae* to which the *S. diversifolia* complex belongs, this does not imply that hybridization has never occurred, as well as influencing the evolutionary history (Ebersbach et al., 2020). It is worth mentioning that the number of samples per population is a limitation of this part of the study. Therefore, we avoided deep conclusions about the detected gene flow events. There have been some assessments about the number of individuals needed for population genomic inference, which show that if a high number of SNPs are considered, only few individuals need to be sampled. For example, Nazareno et al. (2017) revealed that a sample size greater than eight individuals had little impact on estimates of genetic diversity within *Amphirrhox longifolia* (Violaceae) populations, when 1000 SNPs or higher were used. His results also showed that even at a very small sample size (i.e., two individuals), accurate estimates of genetic diversity can be obtained with a large number of SNPs (≥1500).

## 5 CONCLUSIONS

In this study, we successfully sequenced the 53 samples based on RAD‐seq technology, including 15 of the 33 described species in the *S. diversifolia* complex. The SNP calling was subsequently performed, and a total of 111,938 high‐quality SNP loci were detected after a series of rigorous screening. We aim to investigate the phylogenetic relationship of representative species of the *S. diversifolia* complex using the SNP dataset. The result of neighbor‐joining (NJ) tree shows that the *S. diversifolia* complex is a paraphyletic group. Despite of some inconsistencies as revealed by genetic structural analysis, clustering results of representative species reconstructed by both NJ and PCA analyses support previous biogeographic and morphological evidences. Also, it was detected that some taxon may have experienced long‐distance gene flow, from *S. implicans* 1 to *S. implicans* 2, *S. diversifolia* and *S. maxionggouensis*, and *S. maxionggouensis* to *S. nigroglandulifera*.

## AUTHOR CONTRIBUTIONS

**Rui Yuan:** Formal analysis (lead); methodology (lead); software (lead); validation (lead); visualization (lead); writing – original draft (lead); writing – review and editing (lead). **Qingbo Gao:** Conceptualization (equal); funding acquisition (equal); project administration (equal). **Shilong Chen:** Conceptualization (equal); funding acquisition (equal); project administration (equal). **Xiaolei Ma:** Supervision (equal); validation (equal). **Jiaxin Li:** Resources (supporting). **Zhilin Feng:** Resources (supporting). **Rui Xing:** Investigation (supporting); resources (supporting).

## CONFLICT OF INTEREST STATEMENT

None declared.

## Supporting information


Figure S1

Table S1

Table S2

## Data Availability

Raw Illumina RAD‐seq reads are available at the NCBI Short Read Archive (SRA, http://www.ncbi.nlm.nih.gov/sra/) in Bioproject PRJNA944377; Genome survey data in the *S. sinomontana* J‐T. Pan & Gornall is available in PRJNA999480.
