# Supplementary material for: Investigation of phylogenetic relationships within Saxifraga diversifolia complex (Saxifragaceae) based on restriction‐site associated DNA sequence markers
Source: Ecol Evol. 2023 Nov 2;13(11):e10675. doi: 10.1002/ece3.10675 (PMC10620575; doi:10.1002/ece3.10675)
Supplement: Supplementary file 1 — Figure S1 [file ECE3-13-e10675-s003.docx]

Investigation of phylogenetic relationships within *Saxifraga* *diversifolia* complex (Saxifragaceae) based on restriction‐site associated DNA sequence markers

Rui Yuan, Jiaxin Li, Xiaolei Ma, Zhilin Feng, Rui Xing, Shilong Chen, Qingbo Gao

(a)


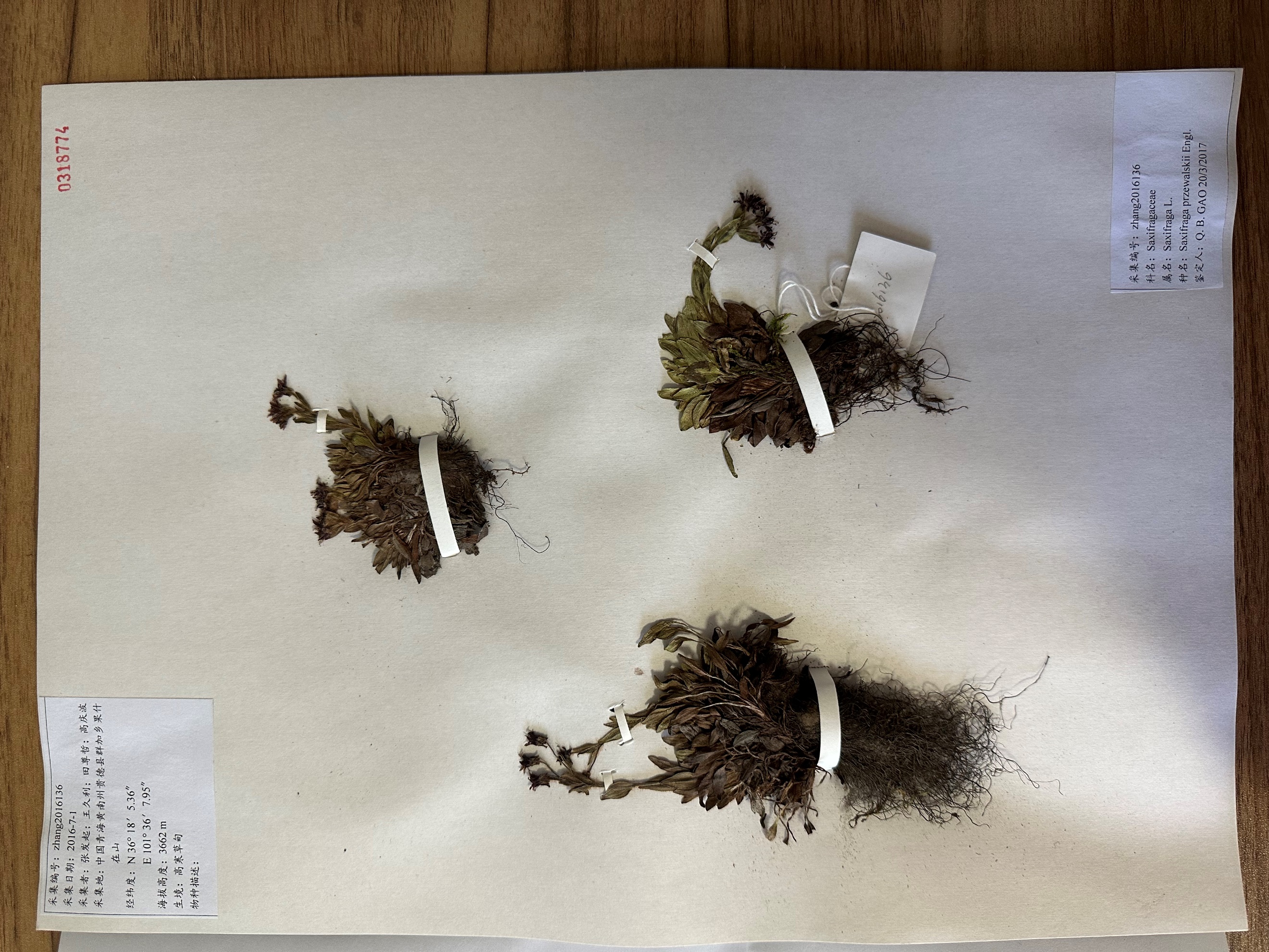


(b)


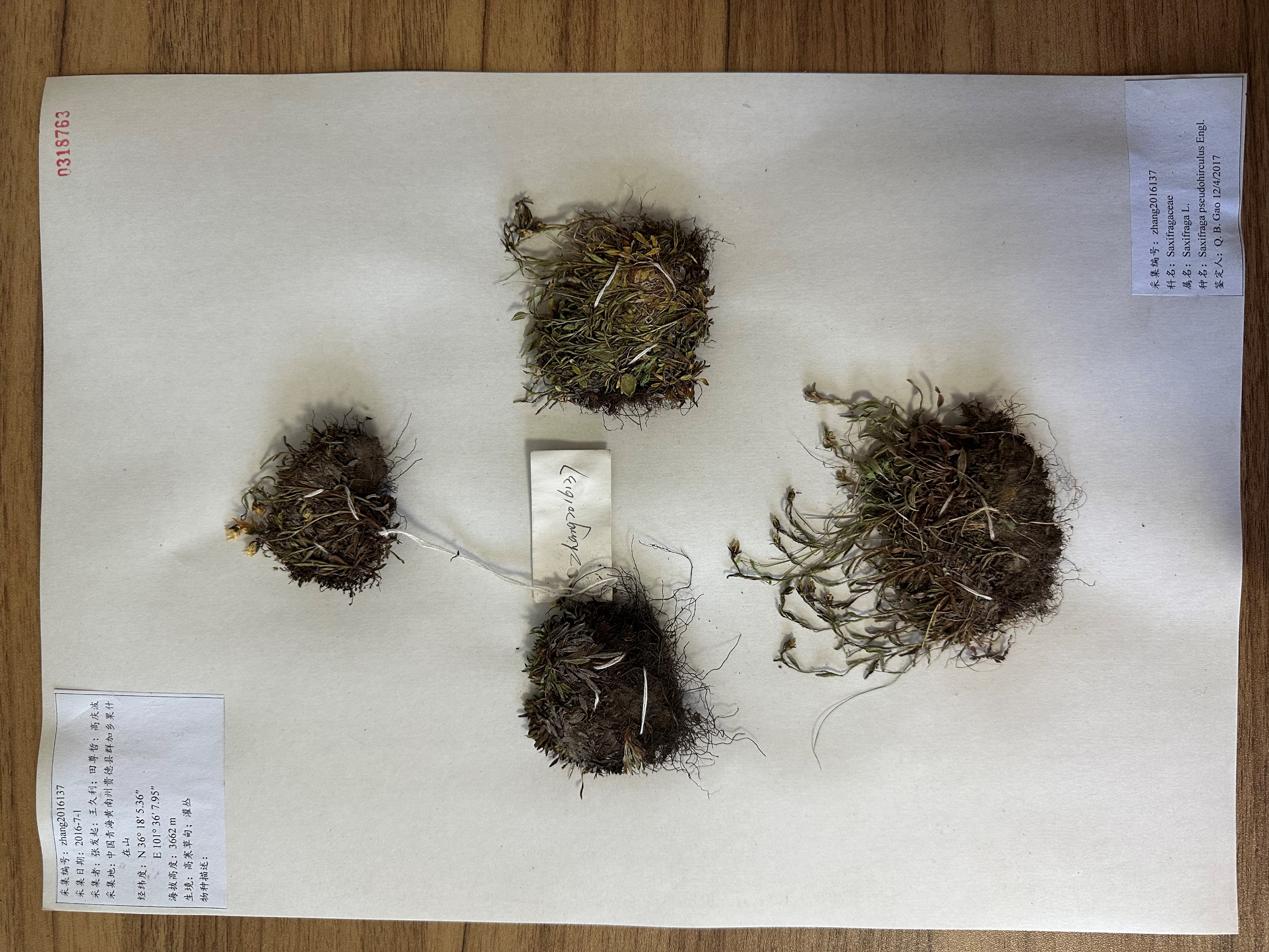


**Appendix Figure S1** Voucher scans of (a) *S. przewalskii* and (b) *S. pseudohirculus*.
