# Supplementary material for: Investigation of phylogenetic relationships within Saxifraga diversifolia complex (Saxifragaceae) based on restriction‐site associated DNA sequence markers
Source: Ecol Evol. 2023 Nov 2;13(11):e10675. doi: 10.1002/ece3.10675 (PMC10620575; doi:10.1002/ece3.10675)
Supplement: Supplementary file 2 — Table S1 [file ECE3-13-e10675-s002.docx]

Investigation of phylogenetic relationships within *Saxifraga* *diversifolia* complex (Saxifragaceae) based on restriction‐site associated DNA sequence markers

Rui Yuan, Jiaxin Li, Xiaolei Ma, Zhilin Feng, Rui Xing, Shilong Chen, Qingbo Gao

**Appendix Table S1** Sequencing data output statistics in the *S. diversifolia* complex

| **Sample** | **Raw Base(bp)** | **Clean Base(bp)** | **Effective Rate(%)** | **Error Rate(%)** | **Q20(%)** | **Q30(%)** | **GC Content(%)** |
| --- | --- | --- | --- | --- | --- | --- | --- |
| *S. bryoides* | 3,056,570,400 | 2,977,529,700 | 97.41 | 0.03 | 97.22 | 92.65 | 37.67 |
| *S. caesia* | 1,597,193,100 | 1,502,820,000 | 94.09 | 0.03 | 97.16 | 92.56 | 39.45 |
| *S. cardiophylla* (1) | 1,368,523,800 | 1,339,928,700 | 97.91 | 0.03 | 96.98 | 92.13 | 38.47 |
| *S. cardiophylla* (2) | 2,711,060,700 | 2,677,163,100 | 98.75 | 0.03 | 97.1 | 92.38 | 38.52 |
| *S. cardiophylla* (3) | 1,784,811,600 | 1,763,965,200 | 98.83 | 0.03 | 96.95 | 92.03 | 38.45 |
| *S. chionophila* | 2,281,628,400 | 2,240,997,900 | 98.22 | 0.03 | 96.97 | 92.08 | 38.08 |
| *S. consanguinea* | 1,222,399,500 | 1,196,896,500 | 97.91 | 0.03 | 96.75 | 91.64 | 37.34 |
| *S. diversifolia* (1) | 8,952,689,700 | 7,827,153,300 | 87.43 | 0.03 | 96.97 | 92.06 | 37.94 |
| *S. diversifolia* (2) | 3,424,306,500 | 2,879,364,900 | 84.09 | 0.03 | 96.97 | 92.04 | 38.01 |
| *S. eglandulosa* (1) | 1,666,697,400 | 1,622,531,100 | 97.35 | 0.03 | 97.09 | 92.4 | 37.21 |
| *S. eglandulosa* (2) | 3,757,179,900 | 3,728,899,200 | 99.25 | 0.03 | 97.5 | 93.22 | 37.67 |
| *S. eglandulosa* (3) | 2,665,926,900 | 2,645,314,500 | 99.23 | 0.03 | 97.47 | 93.12 | 37.53 |
| *S. egregioides* (1) | 1,160,834,400 | 1,145,198,700 | 98.65 | 0.03 | 96.95 | 92.11 | 38.62 |
| *S. egregioides* (2) | 1,430,184,300 | 1,412,722,200 | 98.78 | 0.03 | 96.93 | 92.06 | 38.47 |
| *S. erectisepala* | 2,327,029,800 | 2,229,861,000 | 95.82 | 0.03 | 97.03 | 92.38 | 37.93 |
| *S. gemmigera* | 2,314,221,600 | 2,242,161,300 | 96.89 | 0.03 | 96.9 | 92.05 | 38.11 |
| *S. gemmipara* | 1,931,211,000 | 1,341,672,900 | 69.47 | 0.03 | 96.45 | 91.2 | 38.12 |
| *S. implicans* (1) | 4,251,889,800 | 4,160,631,300 | 97.85 | 0.03 | 97.22 | 92.59 | 38.34 |
| *S. implicans* (2) | 6,096,437,100 | 5,970,255,900 | 97.93 | 0.03 | 97.3 | 92.81 | 38.85 |
| *S. implicans* (3) | 4,911,356,700 | 4,845,822,900 | 98.67 | 0.03 | 97.31 | 92.8 | 38.45 |
| *S. implicans* (4) | 3,811,773,900 | 3,769,550,100 | 98.89 | 0.03 | 97.12 | 92.44 | 38.82 |
| *S. insolens* (1) | 1,318,254,000 | 1,293,569,100 | 98.13 | 0.03 | 97.16 | 92.53 | 38.21 |
| *S. insolens* (2) | 1,306,078,800 | 1,273,615,500 | 97.51 | 0.03 | 97.2 | 92.63 | 38.55 |
| *S. insolens* (3) | 4,688,517,000 | 4,633,102,200 | 98.82 | 0.03 | 97.06 | 92.31 | 38.87 |
| *S. kingdonii* | 1,055,962,800 | 1,041,601,800 | 98.64 | 0.03 | 97.67 | 93.45 | 37.61 |
| *S. maxionggouensis* (1) | 2,517,653,400 | 2,473,525,500 | 98.25 | 0.03 | 97.13 | 92.41 | 39.21 |
| *S. maxionggouensis* (2) | 1,565,553,900 | 1,543,369,200 | 98.58 | 0.03 | 97.05 | 92.23 | 38.89 |
| *S. maxionggouensis* (3) | 2,319,559,200 | 2,234,319,000 | 96.33 | 0.03 | 97.09 | 92.38 | 38.43 |
| *S. maxionggouensis* (4) | 1,417,735,500 | 1,399,389,600 | 98.71 | 0.03 | 97.18 | 92.47 | 38.82 |
| *S. moorcroftiana* | 2,818,828,800 | 2,753,572,200 | 97.68 | 0.03 | 97.02 | 92.15 | 37.53 |
| *S. moschata* | 2,915,144,700 | 2,859,841,200 | 98.1 | 0.03 | 97.27 | 92.72 | 39.27 |
| *S. nigroglandulifera* (1) | 1,693,201,200 | 1,670,312,100 | 98.65 | 0.03 | 97.11 | 92.35 | 38.5 |
| *S. nigroglandulifera* (2) | 2,722,576,800 | 2,677,685,400 | 98.35 | 0.03 | 97.07 | 92.27 | 38.91 |
| *S. nigroglandulifera* (3) | 4,609,691,400 | 4,543,872,000 | 98.57 | 0.03 | 97.19 | 92.59 | 38.25 |
| *S. nigroglandulifera* (4) | 5,733,522,000 | 5,647,364,400 | 98.5 | 0.03 | 97.22 | 92.69 | 38.34 |
| *S. parnassifolia* | 1,748,239,800 | 1,701,803,400 | 97.34 | 0.03 | 96.75 | 91.55 | 38.48 |
| *S. paniculata* | 2,439,400,500 | 2,380,444,500 | 97.58 | 0.03 | 97.15 | 92.49 | 39.48 |
| *S. pardanthina* (1) | 1,941,521,700 | 1,924,841,400 | 99.14 | 0.03 | 96.91 | 92 | 38.64 |
| *S. pardanthina* (2) | 2,006,651,100 | 1,965,281,400 | 97.94 | 0.03 | 97.06 | 92.25 | 38.59 |
| *S. pardanthina* (3) | 2,424,474,900 | 2,394,011,100 | 98.74 | 0.03 | 97.1 | 92.36 | 38.56 |
| *S. pardanthina* (4) | 2,345,067,000 | 2,307,410,400 | 98.39 | 0.03 | 97.03 | 92.3 | 38.91 |
| *S. pratensis* (1) | 1,526,764,800 | 1,418,944,200 | 92.94 | 0.03 | 96.8 | 91.63 | 38.36 |
| *S. pratensis* (2) | 1,212,514,500 | 1,195,779,000 | 98.62 | 0.03 | 97.1 | 92.36 | 38.71 |
| *S. pratensis* (3) | 1,285,445,400 | 1,257,937,200 | 97.86 | 0.03 | 97.06 | 92.24 | 38.63 |
| *S. przewalskii* | 1,879,479,900 | 1,812,600,300 | 96.44 | 0.03 | 96.75 | 91.77 | 38.19 |
| *S. pseudohirculus* | 1,738,287,000 | 1,682,444,100 | 96.79 | 0.03 | 96.91 | 92.03 | 38.34 |
| *S. rotundifolia* | 7,774,838,700 | 7,356,172,800 | 94.62 | 0.03 | 97.01 | 92.02 | 37.39 |
| *S. stellariifolia* (1) | 1,179,132,000 | 1,163,075,400 | 98.64 | 0.03 | 97.12 | 92.37 | 38.49 |
| *S. stellariifolia* (2) | 1,540,637,700 | 1,518,884,400 | 98.59 | 0.03 | 97 | 92.12 | 38.26 |
| *S. stellariifolia* (3) | 2,204,594,400 | 2,169,068,700 | 98.39 | 0.03 | 96.75 | 91.56 | 38.65 |
| *S. stellariifolia* (4) | 3,283,506,900 | 3,242,700,300 | 98.76 | 0.03 | 97.09 | 92.29 | 38.37 |
| *S. subaequifoliata* | 2,956,608,300 | 2,918,772,300 | 98.72 | 0.03 | 97.14 | 92.52 | 38.45 |
| *S. umbellulata* | 1,503,562,500 | 1,454,421,000 | 96.73 | 0.03 | 96.83 | 92 | 38.66 |

Note: the number following species name denotes the count of individuals
