# Supplementary material for: Investigation of phylogenetic relationships within Saxifraga diversifolia complex (Saxifragaceae) based on restriction‐site associated DNA sequence markers
Source: Ecol Evol. 2023 Nov 2;13(11):e10675. doi: 10.1002/ece3.10675 (PMC10620575; doi:10.1002/ece3.10675)
Supplement: Supplementary file 3 — Table S2 [file ECE3-13-e10675-s001.docx]

Investigation of phylogenetic relationships within *Saxifraga* *diversifolia* complex (Saxifragaceae) based on restriction‐site associated DNA sequence markers

Rui Yuan, Jiaxin Li, Xiaolei Ma, Zhilin Feng, Rui Xing, Shilong Chen, Qingbo Gao

**Appendix Table S2** Sequencing depth and coverage statistics

| **Sample** | **Clean_reads** | **mapped_reads** | **mapping_rate** | **Average_depth** | **Coverage_1X** |
| --- | --- | --- | --- | --- | --- |
| *S. bryoides* | 19850198 | 3884570 | 19.57% | 7.04 | 3.51% |
| *S. caesia* | 10018800 | 2458210 | 24.54% | 4.1 | 4.07% |
| *S. cardiophylla* (1) | 8932858 | 7839859 | 87.76% | 6.04 | 11.08% |
| *S. cardiophylla* (2) | 17847754 | 15698204 | 87.96% | 9.46 | 14.77% |
| *S. cardiophylla* (3) | 11759768 | 10293699 | 87.53% | 7.5 | 12.00% |
| *S. chionophila* | 14939986 | 3251133 | 21.76% | 13.37 | 1.54% |
| *S. consanguinea* | 7979310 | 4613411 | 57.82% | 9.08 | 3.83% |
| *S. diversifolia* (1) | 52181022 | 46020130 | 88.19% | 18.08 | 20.88% |
| *S. diversifolia* (2) | 19195766 | 16551935 | 86.23% | 9.48 | 14.44% |
| *S. eglandulosa* (1) | 10816874 | 9381144 | 86.73% | 8.16 | 10.02% |
| *S. eglandulosa* (2) | 24859328 | 21356927 | 85.91% | 14.7 | 13.06% |
| *S. eglandulosa* (3) | 17635430 | 15423847 | 87.46% | 11.79 | 10.91% |
| *S. egregioides* (1) | 7634658 | 6757962 | 88.52% | 6.48 | 8.96% |
| *S. egregioides* (2) | 9418148 | 8443274 | 89.65% | 6.77 | 10.92% |
| *S. erectisepala* | 14865740 | 13132474 | 88.34% | 9.31 | 12.55% |
| *S. gemmigera* | 14947742 | 9343704 | 62.51% | 11 | 6.40% |
| *S. implicans* (1) | 27737542 | 24619980 | 88.76% | 13.94 | 16.09% |
| *S. implicans* (2) | 39801706 | 35283893 | 88.65% | 16.75 | 19.17% |
| *S. implicans* (3) | 32305486 | 28862078 | 89.34% | 14.18 | 18.80% |
| *S. implicans* (4) | 25130334 | 22152595 | 88.15% | 13.29 | 15.40% |
| *S. insolens* (1) | 8623794 | 7626418 | 88.43% | 6.18 | 10.80% |
| *S. insolens* (2) | 8490770 | 7290963 | 85.87% | 6.75 | 9.34% |
| *S. insolens* (3) | 30887348 | 26814836 | 86.81% | 15.48 | 15.29% |
| *S. kingdonii* | 6944012 | 6071052 | 87.43% | 6.41 | 7.49% |
| *S. maxionggouensis* (1) | 16490170 | 14467230 | 87.73% | 9.49 | 13.45% |
| *S. maxionggouensis* (2) | 10289128 | 9049219 | 87.95% | 7.29 | 10.80% |
| *S. maxionggouensis* (3) | 14895460 | 12965607 | 87.04% | 9.36 | 12.27% |
| *S. maxionggouensis* (4) | 9329264 | 8221949 | 88.13% | 7.24 | 9.88% |
| *S. moorcroftiana* | 18357148 | 15944333 | 86.86% | 11.14 | 11.55% |
| *S. moschata* | 19065608 | 3827173 | 20.07% | 9.14 | 2.51% |
| *S. nigroglandulifera* (1) | 11135414 | 9715364 | 87.25% | 8.47 | 9.88% |
| *S. nigroglandulifera* (2) | 17851236 | 15553461 | 87.13% | 10.43 | 13.23% |
| *S. nigroglandulifera* (3) | 30292480 | 26105171 | 86.18% | 16.33 | 14.04% |
| *S. nigroglandulifera* (4) | 37649096 | 32491305 | 86.30% | 19.66 | 14.47% |
| *S. parnassifolia* | 11345356 | 9222459 | 81.29% | 7.22 | 10.27% |
| *S. paniculata* | 15869630 | 3219813 | 20.29% | 6.49 | 3.21% |
| *S. pardanthina* (1) | 12832276 | 11372954 | 88.63% | 8.48 | 11.91% |
| *S. pardanthina* (2) | 13101876 | 11529295 | 88.00% | 8.91 | 11.48% |
| *S. pardanthina* (3) | 15960074 | 14117182 | 88.45% | 9.88 | 12.79% |
| *S. pardanthina* (4) | 15382736 | 13554626 | 88.12% | 9.6 | 12.62% |
| *S. pratensis* (1) | 9459628 | 8072272 | 85.33% | 6.9 | 9.71% |
| *S. pratensis* (2) | 7971860 | 6921758 | 86.83% | 6.48 | 9.14% |
| *S. pratensis* (3) | 8386248 | 7314703 | 87.22% | 6.54 | 9.63% |
| *S. przewalskii* | 12084002 | 10984931 | 90.90% | 7.09 | 14.00% |
| *S. pseudohirculus* | 11216294 | 9796305 | 87.34% | 7.13 | 11.91% |
| *S. rotundifolia* | 49041152 | 13085806 | 26.68% | 9.46 | 4.70% |
| *S. stellariifolia* (1) | 7753836 | 6758169 | 87.16% | 5.98 | 9.78% |
| *S. stellariifolia* (2) | 10125896 | 8795791 | 86.86% | 7.31 | 10.66% |
| *S. stellariifolia* (3) | 14460458 | 12526715 | 86.63% | 8.88 | 12.69% |
| *S. stellariifolia* (4) | 21618002 | 19288696 | 89.23% | 11.57 | 15.17% |
| *S. subaequifoliata* | 19458482 | 17183330 | 88.31% | 11.33 | 13.59% |
| *S. umbellulata* | 9696140 | 5966508 | 61.53% | 9.11 | 4.66% |
| *S. gemmipara* | 8944486 | 4576663 | 51.17% | 3.97 | 7.59% |

Note: the number following species name denotes the count of individuals
